# Delayed Mental Nerve Neuralgia following Chin Augmentation

**DOI:** 10.1155/2013/860634

**Published:** 2013-01-20

**Authors:** Ineke Wever, Sang Hwang, Sim Choroomi, William Mooney

**Affiliations:** Division of Surgery, Department of Otolaryngology, The Prince of Wales Hospital and Community Health Services, Barker Street, Randwick, NSW 2031, Australia

## Abstract

This paper describes a case of mental nerve neuralgia following a traumatic dislodgement of a chin implant ten months after surgery. Our case is unusual, both in the specific complication and the patients' atypical representation—delayed and initially without mention of trauma. To the authors' knowledge, this case has not been reported previously in the literature. We review the complications of chin augmentation and the techniques for fixation and discuss implications for the preoperative disclosure with patients.

## 1. Introduction

Mental nerve injury can occur as a consequence of the various chin augmentation techniques to correct microgenia or “small chin.” The mental nerve is particularly vulnerable during more invasive procedures such as horizontal advancement osteotomy. Augmentation using an implant is considered to be a safer simpler procedure with fewer complications. Mental nerve injury occurs occasionally; other more common complications include migration/displacement, tissue reaction, lower lip incompetence, patient dissatisfaction, and infection, which may occur much later with chin than other facial implants [1]. We review the unique case of delayed mental nerve neuralgia that followed traumatic dislodgement of chin implant ten months after surgery.

## 2. Case Presentation

**Figure 1:**
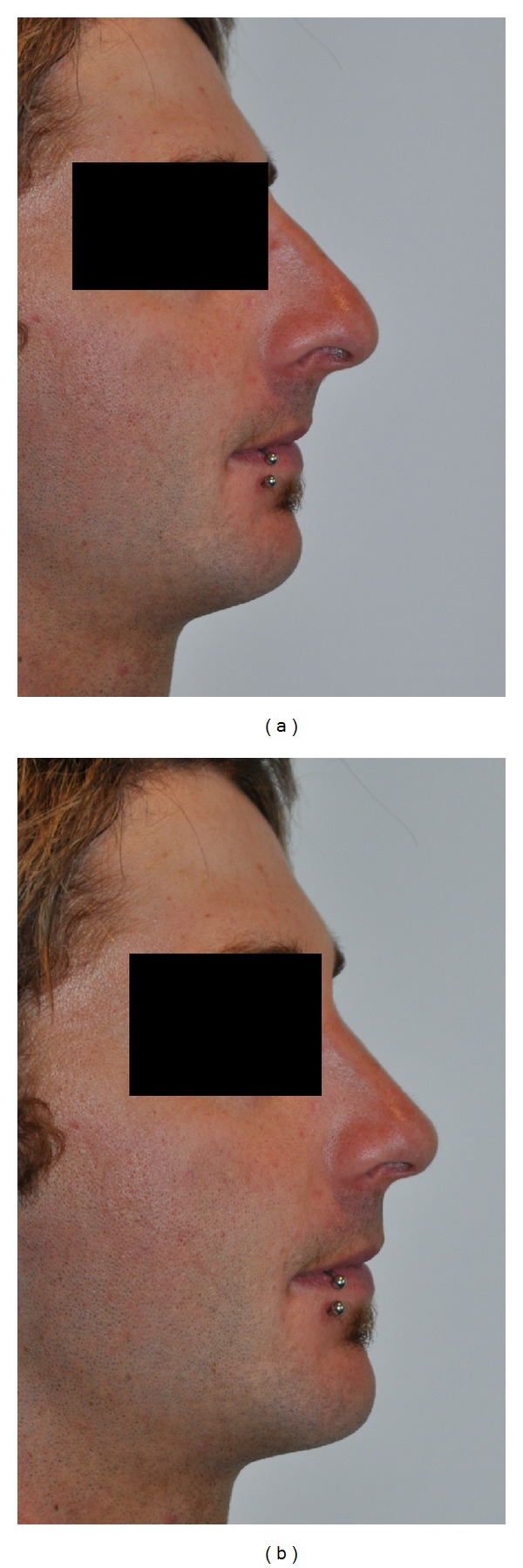
(a) Photograph of the patient preoperatively. (b) Using visual photographic reconstruction to demonstrate facial augmentation—planned nasal dorsal hump reduction and augment chin into alignment with mid facial plane.

**Figure 2:**
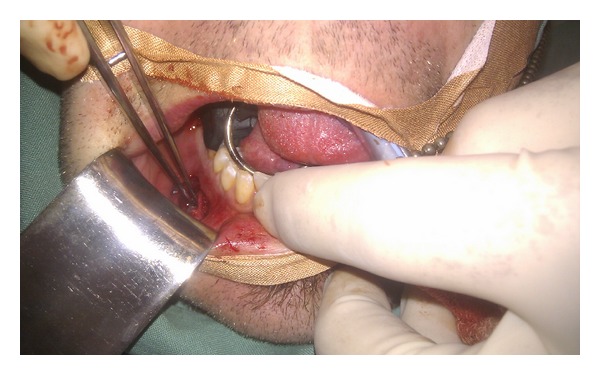
Photograph showing intraoperative findings of implant pressing on mental nerve.

**Figure 3:**
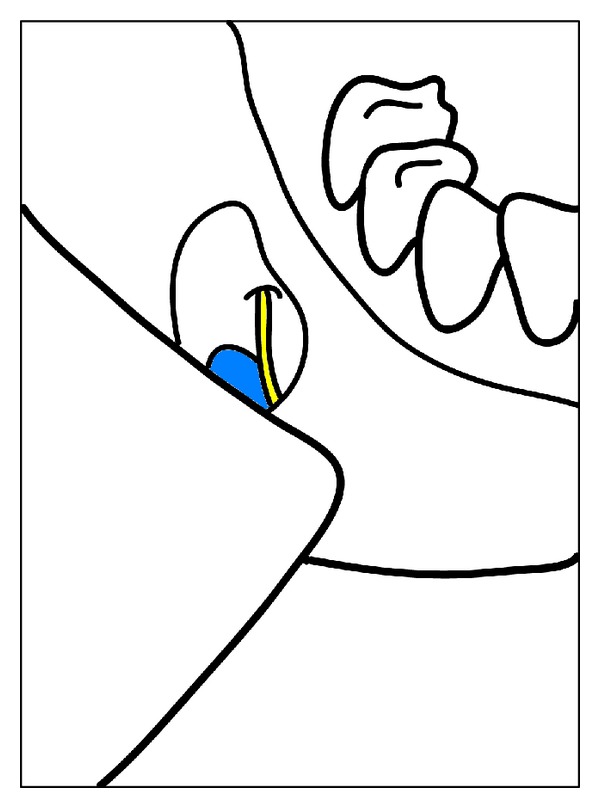
Diagrammatic representation of intraoperative findings of chin implant (blue) inferior to mental nerve (yellow).

**Figure 4:**
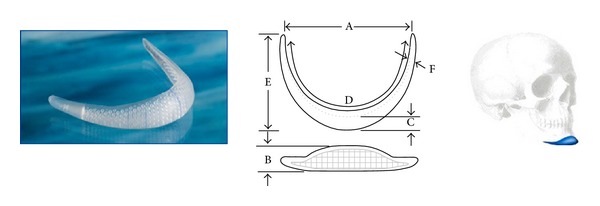
Implantech Conform extended anatomical chin implant used.

A 27-year-old male presented with a functional and cosmetic concerns regarding his nose. His cosmetic concerns included a prominent dorsal hump and overprojection. He had a history of traumatic rugby impact on the nose but had never sought treatment. Functionally, he noticed nasal obstruction, worse at night. After confirming septal deviation with nasopharyngoscopy and proceeding to preliminary photographic visual planning, both the patient and the surgeon noted that a balanced facial aesthetic would require a specific correction of the patients microgenia to the mid facial plane (see Figure 1). Chin augmentation with an Implantech Conform extended anatomical chin implant was agreed as a simultaneous procedure to overcome his chin recession (see Figure 4). Septorhinoplasty proceeded uneventfully and the chin augmentation via a transoral approach was undertaken successfully with care to identify and preserve mental nerves. Fixation for the implant was achieved by creating a snug pocket for the implant and two-layered closure. The patient recovered well in the immediate postoperative period. He represented ten months postoperatively with dysesthesia over the right lower lip, a burning shooting pain, and had noticed a small lump intraorally along the right lower gingivobuccal sulcus. One month prior to this representation, the patient had undergone wisdom teeth extraction and the ensuing dysesthesia over the mental nerve distribution was thought by the surgeon to be temporally related to the dental procedure. The intraoral lesion was planned for biopsy under general anaesthetic. Intraoperatively, the right mandibular submucosal lesion planned for biopsy was confirmed to be the migrated chin implant impinging on the mental nerve (see Figures 2 and 3). Superior displacement of the chin implant was causing mental nerve irritation by elevating the mental nerve shortly after exiting the mental foramen. Shortening of the implant was undertaken by one centimetre and a steroid injection also administered intraoperatively to relieve nerve compression. The patient recovered well postoperatively and experienced complete resolution of his symptoms. Only postoperatively did the patient recall a history of trauma to the area approximately one month earlier: his daughter's head contacted the right mandibular region. This impact was posited to be the time of the implant migration.

## 3. Discussion

Chin augmentation or augmentation mentoplasty with synthetic implants was first introduced in the 1950s and has become an increasingly popular cosmetic procedure for chin retrusion or microgenia [2, 3]. This procedure involves the placement of an implant in the subperiosteal pocket with the aim of improving chin projection and/or augmenting the chin [1].

Complications of chin augmentation include infection, malposition of the implant (leading to patient dissatisfaction), and extrusion of the implant intraorally [1, 4]. A case series of 324 chin implants by Godin et al. found an infection rate of 0.62%, while another case series of 125 consecutive patients by Aynehchi et al. reported no infections [1, 5]. There were no cases of chin implant migration in both case series.

Chin augmentation with synthetic implants is a less complicated procedure than genioplasty which requires an osteotomy and replating and consequently is associated with less surgical morbidity [6]. Mental nerve injury and neuralgia is reported as high as 10% when an osteotomy is performed as anatomical variations of the inferior alveolar canal and mental foramen confer risk to the nerve [7, 8]. Synthetic chin implant augmentation mitigates this risk as the mental nerve can be identified and protected during the surgical procedure.

Both transoral and external approaches have been described for the insertion of chin implants. A preference for the transoral approach is evident in the literature as it avoids an external scar, which had been reported to cause scar alopecia in males [1]. The complication rates for both approaches are comparable, although some authors state a lower infection and malposition rate with the external approach [4, 9].

The surgical technique involves the creation of an optimally sized subperiosteal pocket in which the chin implant is placed, minimising the risk of implant migration [1]. Although additional fixation techniques with sutures or screws can be used, this introduces a greater risk of damage to the mental nerve and mentalis muscle and is not necessary as a primary procedure [1].

Traumatic dislodgement and migration of chin implants is an unexpected complication and not normally reported in long-term morbidity studies. Our case highlights an unusual migration and as previously noted in the literature, an external submental approach to resite and/or secure the chin implant is recommended [6, 9].

There have been no prior reported cases of mental nerve compression secondary to traumatic chin implant migration in the recent literature. This case highlights a potential complication to discuss with patients who participate in high risk activities for facial trauma such as contact sports. Mental nerve compression causes distressing symptoms for the patient and should be discussed when considering elective chin augmentation.
